# Supplementary material for: Hunger and Health: Taking a Formative Approach to Build a Health Intervention Focused on Nutrition and Physical Activity Needs as Perceived by Stakeholders
Source: Nutrients. 2021 May 10;13(5):1584. doi: 10.3390/nu13051584 (PMC8151779; doi:10.3390/nu13051584)
Supplement: Supplementary file 1 [file nutrients-13-01584-s001.zip › nutrients-1175436-supplementary.pdf]

nutrients MDPI

Supplementary

Hunger and Health: Taking a Formative Approach to Build a Health Intervention Focused on Nutrition and Physical Activity Needs as Perceived by Stakeholders

Kelsey Fortin * and Susan Harvey

Supplementary 1

Shaping a Food Pantry Health Intervention- Client Survey

Thank you for your willingness to participate in this study. Your feedback and responses are important to us. If youhave any questions regarding this survey, please feel free to ask one of the researchers in the room.

<u>Health Status</u>

1. **Have you ever been diagnosed with any of the following conditions?**

	Yes	No	Never Tested
Diabetes	1	2	3
High blood pressure	1	2	3
High cholesterol	1	2	3
Heart disease	1	2	3
Metabolic syndrome	1	2	3
Other (Please list):			

2. **Has anyone in your household (other than yourself) ever been diagnosed with any of the followingconditions?**

	Yes	No	Not Sure
Diabetes	1	2	3
High blood pressure	1	2	3
High cholesterol	1	2	3
Heart disease	1	2	3
Metabolic syndrome	1	2	3
Other (Please list):			

3. **Would you say that in general your health is: (Please check only <u>ONE</u> response).**

_______Excellent _______Very Good _______Good

_______Fair _______Poor

4. **When is the last time you have been to the Doctor (Please check only <u>ONE</u> response)?**

_______Within the last 1 to 3 months ______4 to 6 months ago

_______More than a year

What was the reason for your visit? ____

5. **Do you currently have health insurance?**

    _______Yes _______No

Nutrition Habits

6. **How many servings of FRUIT do you usually eat PER DAY? [Think of a serving of fruit as being about 1 medium piece, or 1 cup of fruit. Please see food models to help you in answering this question]. Pleasecheck only ONE response**

    _______0 to 1 serving _______2 to 3 servings _______More than 3 servings

7. **How many servings of VEGETABLES do you usually eat PER DAY? [Think of a serving of vegetables as being about 2 cups of raw leafy vegetables, or 1 cup of other raw or cooked vegetables. Please see foodmodels to help you in answering this question]. Please check only ONE response.**

    _______0 to 1 serving _______2 to 3 servings _______More than 3 servings

8. **How many servings of PROTEIN do you usually eat PER DAY? [Think of one serving of protein as being 1egg; 3 ounces of meat, fish, poultry (size of a deck of cards); ½ cup of beans (one fist); 2 tablespoons of peanut butter (two thumb tips). Please see food models to help you in answering this question]. Please check only ONE response.**

    _______0 to 1 serving _______2 to 3 servings _______More than 3 servings

9. **How many servings of DAIRY do you usually eat PER DAY? [Think of one serving of dairy as being one cup of milk or yogurt; 2 ounces of cheese (1 slice, 1 stick, three dice if cubed). Please see food models tohelp you in answering this question]. Please check only ONE response.**

    _______0 to 1 serving _______2 to 3 servings _______More than 3 servings

10. **How many servings of GRAINS do you usually eat PER DAY? [Think of one serving of grains as being 1slice of bread (size of a CD case); ½ cup cooked pasta, rice, cereal; 1 6-inch tortilla (taco size). Please see food models to help you in answering this question). Please check only ONE response.**

    _______0 to 1 serving _______2 to 3 servings _______More than 3 servings

11. **How often do you choose WHOLE GRAIN options? Please check only ONE response.**

    _______Less than 50% of the time _______More than 50% of the time

12. **How often do you typically get your food from a food pantry? Please check only ONE response.**

    _______Less than once a month _______Once a month _______2 to 3 a month

    _______Once a week _______More than once a week

13. **The following reasons REGULARLY prevent me from eating healthy meals.**

Healthy foods are too expensive.	Yes	No
I don’t know enough about healthy cooking.	Yes	No
Healthy foods spoil too quickly.	Yes	No
Healthy foods take too much time to cook/prepare.	Yes	No
Healthy foods don’t taste good to me and/or my family.	Yes	No
I don’t know enough about general nutrition to make healthy meals.	Yes	No
I don’t know how to choose and store fresh produce.	Yes	No

I or someone in my household has health conditions that restrict certain foods or food groups.	Yes	No
I don't have the right utensils or appliances (e.g., stove) to prepare them.	Yes	No
I can't afford the utility bills (e.g., electricity) that result from cooking them.	Yes	No

Other (Please list):

Physical Activity Habits

14. **How many minutes PER WEEK do you participate in recreational physical activity (Examples: walking, jogging, swimming, aerobics)? Please check only ONE response.**

_______0 to 60 minutes _______60 to 90 minutes

_______90 to 120 minutes _______120 to 150 minutes

_______More than 150 minutes

15. **When you engage in physical activity, how often do you reach the point of sweating? Please check only ONE response.**

_______Never _______Rarely _______Often

_______Always _______I don't engage in physical activity

16. **What is your activity level at work? Please check only ONE response.**

_______Low (sitting at desk) _______Low-medium (mostly sitting combined with walking/lifting/standing) _______Medium (equal combination of sitting, walking,lifting, and/or standing)

_______Medium-high (most activities involve walking, standing, lifting, and/or walking) _______High (standing, heavylifting, walking) _______I currently do not work

17. **The following reasons REGULARLY prevent me from being physically active.**

Physical activity is too expensive.	Yes	No
I don't know enough about physical activity.	Yes	No
I don't have enough time to engage in physical activity.	Yes	No
I or someone in my household has health conditions that restricts my physical activity.	Yes	No
Physical activity isn't fun to me and/or my family.	Yes	No
I don't have access to a facility to engage in physical activity	Yes	No
I have other priorities that are more important than being physically active.	Yes	No
My job is physically demanding.	Yes	No

Other (Please list): ____

Demographics

18. Which would you say best represents your gender? Please check only ONE response

_______Male _______Female

_______Trans Gendered _______Prefer not to answer

19. What is your age? ____

20. Which would you say best represents your race/ethnicity? Please check only ONE response.

_______Caucasian/White (not Hispanic) _______African American/Black

_______Hispanic/Latino _______Asian

_______Native American _______Mixed

Other (Please describe): ____

21. What is your estimated annual household income? Please check only ONE response.

_______Under $10,000 _______$10,000 to $24,999

_______$25,000 to $49,999 _______$50,000 to $74,999

_______$75,000 to $99,999 _______$100,000 or more

22. Are you working? Please check only ONE response.

_______Full-time _______Part-time _______Unemployed, currently seeking work

_______Unemployed, notcurrently seeking work _______Student _______Retired

23. What is the HIGHEST level of school you completed? Please check only ONE response.

_______5th grade _______8th grade _______Some high school

_______High school graduate or GED _______Some college _______Associate's degree

_______Bachelor's degree _______Master's degree _______Professional/doctoral degree

24. Including yourself, how many individuals live in your household? ______________

25. How many individuals UNDER 18 live in your household? ____________________

26. Do you have access to a computer AT HOME? _______Yes _______No

27. Do you have access to the internet AT HOME? _______Yes _______No

28. What is the best way to share information about programs and resources offered for nutrition and physical activity? (check all that apply)

_______Just Food Website

_______Just Food Facebook page

_______Just Food Instagram Page

_______From a volunteer/staff member

_______From another Just Food client

_______Flyer

_______I have not heard of any programs

Other, please specify: ______________________________

Supplementary 2

Shaping a Food Pantry Health Intervention- Client

Interview Questions

Nutrition

1. What guides you to make your food choices while you shop for food?
2. What guides your decision making for meals (prompt: how often do you eat out vs. cooking at home, preparing and cooking meals)?
3. What are some of the challenges and barriers to choosing and cooking healthy options?
4. What are some topics and/or skills you would be interested in learning that could help you be more successful with health eating?

Physical Activity

5. Talk about your current physical activity level (prompt: how often, how long, what types of activities)
6. What are some of the challenges and barriers to engaging in regular physical activity? (*Prompts: working longhours, standing a lot, health conditions, time, childcare*)
7. What are some topics and/or skills you would be interested in learning that could help you to be moresuccessful in engaging in regular physical activity?

Health Status

8. What current health issues are you and/or members of your household facing? Does this affect the types offood you select?
9. How do the health issues you have described impact your lifestyle, such as preparing and cooking meals orengag-ing in regular physical activity?

Health Programming

We are considering creating a health intervention program at the food pantry. The purpose of the program will beto have someone clients can meet with regularly to discuss topics in nutrition, physical activity, and health, as a part of a holistic health intervention.

10. What do you think are some critical characteristics of this program? (Prompt: how often meetings are, time ofday, days of the week, how long, educator characteristics, location, electronic Vs. in-person)
11. What do you think are important topics that should be covered during education sessions?
12. What are some of the challenges and barriers to participating in this type of programs?
13. How would you like to be informed about this program?
14. Other thoughts or feedback?

Supplementary 3

Shaping a Food Pantry Health Intervention- Staff/volunteer Survey

Thank you for your willingness to participate in this study. Your feedback and responses are important tous. If you have any questions regarding this survey, please feel free to ask one of the researchers in the room.

Health

1. **How often do you engage with clients about the following health related topics?**

	**Often**	**Sometimes**	**Never**
Diabetes	1	2	3
High blood pressure	1	2	3
High cholesterol	1	2	3
Heart disease	1	2	3
Metabolic syndrome	1	2	3
Health insurance	1	2	3
Local low-costhealth service	1	2	3
Other (Please list):			

2. **How capable to do you feel about answering questions asked related to health topics?**

_______Very capable _______Somewhat cable _______Not capable

3. **Which of the following topics would you be interested in receiving additional staff training in (check ALL that apply)**

_______Diabetes _______Blood pressure

_______Cholesterol _______Heart Disease

_______Metabolic syndrome _______Health insurance

_______Local low-cost health service _______I would not like additional training

_______Other (Please list): ____

Nutrition

4. **How often do you engage with clients about the following nutrition related topics?**

	**Often**	**Sometimes**	**Never**
Cost of food	1	2	3
Storage of food	1	2	3
General healthy eating	1	2	3
Building health meals	1	2	3
Use of unusual pantryitems (e.g., eggplant)	1	2	3
Recipe make overs (e.g., healthy ramen)	1	2	3
Quick meal options	1	2	3
Food restrictions	1	2	3
Other (Please list):			

5. **How capable to do you feel about answering questions asked related to nutrition?**

_______Very capable _______Somewhat cable _______Not capable

6. **Which of the following topics would you be interested in receiving additional staff training in (check ALL that apply)**

_______Cost of food

_______Storage of food

_______General healthy eating

_______Building health meals

_______Use of unusual pantry items (e.g., eggplant)

_______Recipe make overs (e.g., healthy ramen)

_______Quick meal options

_______Food restrictions

_______I would not like additional training

Other (Please list): ____

Physical Activity

7. **How often do you engage with clients about the following physical activity related topics?**

	Often	Sometimes	Never
Cost of physicalactivity	1	2	3
General physical activity guidelines	1	2	3
Lack of time to engagein physical activity	1	2	3
Physical demands ofoccupation	1	2	3
Lack of access tofacilities	1	2	3
Local low-cost physical activity services	1	2	3
Physical limitations	1	2	3
Other (Please list):			

8. **How capable to do you feel about answering questions asked related to Physical activity?**

_______Very capable _______Somewhat cable _______Not capable

9. **Which of the following topics would you be interested in receiving additional staff training in (check ALL that apply)**

_______Cost of physical activity

_______General physical activity guidelines

_______Lack of time to engage in physical activity

_______Physical demands of occupation

_______Lack of access to facilities

_______Local low-cost physical activity service

_______Physical limitations

_______I would not like additional training

Other (Please list): ____

Demographics

10. **Which would you say best represents your gender? Please check only ONE response**

_______Male _______Female

_______Trans Gendered _______Prefer not to answer

11. **What is your age? ____**

12. **Which would you say best represents your race/ethnicity? Please check only ONE response.**

_______Caucasian/White (not Hispanic) _______African American/Black

_______Hispanic/Latino _______Asian

_______Native American _______Mixed

Other (Please describe): ____

13. **Which of the following best describes you employment status at Just Food? Please check only ONE response (Staffonly).**

_______Full-time _______Part-time _______AmeriCops/Vista

14. **What is the HIGHEST level of school you completed? Please check only ONE response.**

_______5th grade	_______8th grade	_______Some high school
_______High school graduate or GED	_______Some college	_______Associate's degree
_______Bachelor's degree	_______Master's degree	_______Professional/doctoral degree

15. **How many years have you been a staff member/volunteer at Just Food (*round to the nearest year*)? _____________**

Supplementary 4

Shaping a Food Pantry Health Intervention- Staff/volunteer

Interview Questions

1. What is your role as a staff member/volunteer at Just Food (prompt: Job duties, years of service, etc.)?
2. What is your interaction with clients like as a staff member/volunteer?
3. What questions do clients most commonly ask about:
    a. Food/food products
    b. Nutrition
    c. Physical activity
    d. Health
    e. Programs/resources offered by Just Food
4. What changes would you make to shopping and the shopping floor to better address these client questions?
5. What are the most important topics within nutrition, physical activity, and health that clients should be addressed in a health intervention program?
6. If additional training was offed for staff/volunteers what do you think the most important topics would berelated to nutrition, physical activity, and health for you to better assist clients?
7. What are some barriers to providing/attending this additional training?
8. Other thoughts or feedback?
